# Wide Band Low Noise Love Wave Magnetic Field Sensor System

**DOI:** 10.1038/s41598-017-18441-4

**Published:** 2018-01-10

**Authors:** Anne Kittmann, Phillip Durdaut, Sebastian Zabel, Jens Reermann, Julius Schmalz, Benjamin Spetzler, Dirk Meyners, Nian X. Sun, Jeffrey McCord, Martina Gerken, Gerhard Schmidt, Michael Höft, Reinhard Knöchel, Franz Faupel, Eckhard Quandt

**Affiliations:** 1 Institute for Materials Science, Kiel University, Kiel, 24143 Germany; 2 Institute of Electrical Engineering and Information Technology, Kiel University, Kiel, 24143 Germany; 3 W.M. Keck Laboratory for Integrated Ferroics, Electrical and Computer Engineering Department, Northeastern University, Boston, Massachusetts 02115 USA

## Abstract

We present a comprehensive study of a magnetic sensor system that benefits from a new technique to substantially increase the magnetoelastic coupling of surface acoustic waves (SAW). The device uses shear horizontal acoustic surface waves that are guided by a fused silica layer with an amorphous magnetostrictive FeCoSiB thin film on top. The velocity of these so-called Love waves follows the magnetoelastically-induced changes of the shear modulus according to the magnetic field present. The SAW sensor is operated in a delay line configuration at approximately 150 MHz and translates the magnetic field to a time delay and a related phase shift. The fundamentals of this sensor concept are motivated by magnetic and mechanical simulations. They are experimentally verified using customized low-noise readout electronics. With an extremely low magnetic noise level of ≈100 pT/$\sqrt{{\rm{Hz}}}$, a bandwidth of 50 kHz and a dynamic range of 120 dB, this magnetic field sensor system shows outstanding characteristics. A range of additional measures to further increase the sensitivity are investigated with simulations.

## Introduction

Magnetic field sensing is an important task for many applications ranging, e.g., from positioning and navigation to electronic stability programs, electrical current sensors to biomagnetic field detection^1–3^. Naturally, the requirements differ and depend on the specific application. Common demands on sensors include ambient temperature operation without cooling, small dimensions necessary for high spatial resolution and/or limited available installation space as well as low energy consumption. Very demanding specifications arise in terms of the dynamic magnetic field range and the frequency bandwidth in case of a current sensor^4^ as well as in a limit of detection (LOD) in the pT/$\sqrt{{\rm{Hz}}}$ to fT$\sqrt{{\rm{Hz}}}$ range in case of sensors for biomagnetic signals^5^. Furthermore, both applications require the detection of DC or very low frequency magnetic fields, which is especially challenging if 1/*f* (*f*: frequency) noise is present. Ultra-low LODs for magnetic field sensing in combination with ambient temperature operation and sufficient spatial resolution have been reported for orthogonal fluxgate magnetometers^6^, sensors employing the giant magnetoimpedance effect^7^, atomic magnetometers^8^, magnetoresistive devices^9^, and, most recently, for sensors using the ΔE-effect^10^. Table 1 summarizes the main important properties of these sensors.

**Table 1:** Overview of compact high resolution magnetometers that operate at ambient temperature. A range above 50 μT enables unshielded operation. Biomagnetic applications require a detection limit below at least 10 pT/$\sqrt{{\rm{Hz}}}$.

	Microwire fluxgate	Giant magnetoimpedance	Atomic magnetometer	Anisotropic magnetoresitive	Resonant ΔE-effect
LOD at 1 Hz	1.5 pT/$\sqrt{{\rm{Hz}}}$ ^6^	3 pT/$\sqrt{{\rm{Hz}}}$ ^7^	10 fT/$\sqrt{{\rm{Hz}}}$ ^8^	1 nT/$\sqrt{{\rm{Hz}}}$ ^9^	300 pT/$\sqrt{{\rm{Hz}}}$ ^10^
Frequency Bandwidth	400 Hz^6^	70 kHz^11^	100 Hz^8^	1 MHz^9^	5 Hz^10^
Range	1000 nT^6^	±100 μT^11^	15 nT^8^	±0.5 mT^9^	>1 μT^10^

Magnetoelastic coupling describes the property of materials to have an interdependency between magnetization and elastic strain. It is commonly known in the form of so-called Joule magnetostriction that causes the humming noise in electrical transformer cores, where the core length changes during the magnetization. In an inverse process (also known as Villari Effect) under mechanical stress, these materials exhibit an additional strain caused by a rotation of the magnetic moments. Such extra strain leads to altered elastic properties proportional to the material’s piezomagnetic coefficient. For high piezomagnetic coefficients, softmagnetic properties in combination with a high magnetostriction are required: thus, the largest piezomagnetic coefficients have been found in amorphous FeGaB^12^ or FeCoSiB^13^ thin films. The phenomenon is commonly known as the ΔE-effect for changes in the Young’s modulus *E* ^14–16^, but other elastic constants are also affected. Although it is well known that the relative effects of other elastic moduli such as the shear modulus *G* are generally more pronounced^17^, most approaches for magnetic sensing have been made using *E*. The ΔE-effect has been used to detune either cantilever^18–20^ or bulk resonators^21^ coated with a magnetic material. Also, surface acoustic waves (SAWs) have been utilized in resonator or delay line configurations^22^. These approaches use a piezoelectric substrate with two interdigital transducer (IDT) electrodes, one to excite and one to receive acoustic waves. If a magnetic film is deposited in-between these two IDT electrodes, changes in the elastic properties of the film influence the wave velocity and alter the delay of a transmitted signal.

There are various kinds of waves that can be excited, and they differ in the type of deformation and velocity due to the different elastic moduli involved. Rayleigh waves are concentrated at the surface with out-of-plane shear and longitudinal deformation. Their amplitude exponentially decays with a penetration depth of about one wavelength. In amorphous magnetic thin films greater than 50 nm thick, the magnetization is usually confined in-plane, which restricts all changes of elastic components to this plane. Consequently, the propagation of Rayleigh waves is only magnetically sensitive to magnetoelastic alterations of *E*. Shear horizontal waves perform a bulk shear motion parallel to the magnetic film and are thus sensitive to changes of *G*. While the wave extends through the whole substrate, the modulus effect occurs only in the magnetic layer on the top and, consequently, has little leverage on the wave velocity. Love waves, in contrast, combine the advantages of both wave types with respect to magnetic field sensing. They are horizontal shear waves that are confined at the surface by a phase velocity gradient towards the surface^23^. Despite these advantages, most studies focus on Rayleigh waves^24,25^, few on shear horizontal waves^26^ and even fewer on Love waves^27^. Although numerous devices with strong coupling of surface waves to magnetic properties have been presented, many were intended to be used as magnetically tunable RF-components^22^, and only a few have been thoroughly analyzed for their potential as magnetic field sensors.

In this paper, the design of a novel Love wave-type magnetic field SAW sensor will be outlined with an emphasis on finite element modeling (FEM) simulations to work out the advantages of using a dedicated guiding layer. Next, the SAW sensor will be characterized electrically and the magnetic properties of the magnetostrictive layer will be investigated and compared with simulations. Then, the sensor will be integrated in a readout circuit, which is tailored to obtain a sensor system, where the intrinsic noise of the sensor dominates. Very promising characteristics of the sensor system in terms of sensitivity, LOD, dynamic range, and bandwidth will be measured and analyzed. Prospects of different measures to further increase the sensitivity and the LOD will be proposed thereafter.

## Sensor Design

**Figure 1:**
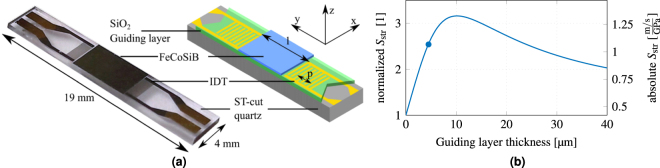
Sensor design and modeled structural sensitivity. (**a**) A Love wave surface acoustic wave sensor based on ST-cut quartz substrate and a 4.5 μm SiO_2_ guiding layer. The 200 nm magnetostrictive material FeCoSiB is deposited between the transducers on top of the delay line with a length *l* of 3.8 mm. The interdigital transducers have a periodicity *p* of 28 μm. (**b**) Simulation of structural sensitivity *S*_str_ for different guiding layer thicknesses and a 200 nm thick magnetostrictive layer. The sensitivity of the fabricated device (indicated by blue dot) is close to the maximum sensitivity at 10 μm.

The presented Love wave sensor is based on a 500 μm ST-cut quartz substrate and is shown in Fig. 1a. The two IDT electrode pairs form a delay line of *l* = 3.8 mm. They are made of 300 nm Au and structured by ion-beam etching to a doublefinger structure of 25 pairs with a periodicity of 28 μm and a finger width of 3.5 μm. A 12 nm Cr layer above and below the Au serves as an adhesion promoter. The propagation direction of the wave is orthogonal to the X-axis of the ST-cut quartz wafer. In this configuration only shear waves are excited^23^. A layer of 4.5 μm thick SiO_2_ is deposited with a PECVD process and covers the IDTs and the delay line, and is angled at both ends to prevent multiple transient signals. The structure acts as a guiding layer and leads to a concentration of the wave energy in the magnetic material. The magnetostrictive material (Fe_90_Co_10_)_78_Si_12_B_10_ is deposited with a thickness of 200 nm by magnetron sputtering on the delay line and structured using a lift-off process. During deposition a magnetic field is applied along the Y-axis to saturate the film and introduce an easy axis of magnetization. Below and above the magnetic layer a 10 nm Ta layer is deposited to promote adhesion and prevent oxidation.

The overall sensitivity S of the sensor is the product of several major contributions that are introduced here and discussed in detail in the following sections. The sensitivity of the magnetic material

1 $${S}_{{\rm{mag}}}=\frac{\partial G}{\partial H}$$

corresponds to the change in the shear modulus *G* in the presence of an external magnetic field *H*. *S*_mag_ depends on the magnetostrictive properties of the material, the orientation of the magnetic easy axis, the directions of the wave propagation, and external magnetic field, respectively. How much the change in shear modulus affects the velocity *v* of the surface wave is expressed by a structural sensitivity

2 $${S}_{{\rm{str}}}=\frac{\partial v}{\partial G}\mathrm{.}$$

*S*_str_ is influenced by the vertical structure of the device that influences the confinement of the wave as well as the contribution of each layer. According to the FEM-simulation results shown in Fig. 1b an even higher factor of 3.16 could be achieved by choosing an 10 μm thick guiding layer, but was not carried out because of process limitations. The chosen thickness of 4.5 μm still increases the structural sensitivity *S*_str_ by a factor of 2.54 to 1.07$\frac{{\rm{m}}/{\rm{s}}}{{\rm{GPa}}}$.

The phase shift per change in wave velocity depends on the geometric sensitivity *S*_geo_

3 $${S}_{{\rm{geo}}}=\frac{\partial \phi }{\partial v}=-\frac{l}{{v}^{2}}\cdot f\cdot 2\pi $$

of the device. A change in wave velocity *v* at the frequency *f* leads to a change of the phase $\phi =\frac{l}{v}\cdot f\cdot 2\pi$ at the end of the delay line with a length *l*. The results of the simulations are a geometric sensitivity of ${S}_{{\rm{geo}}}=-11.3\frac{^\circ {\rm{s}}}{{\rm{m}}}$. In combination, these three contributions lead to the overall sensitivity

4 $$S=\frac{\partial \phi }{\partial H}=\frac{\partial G}{\partial H}\cdot \frac{\partial v}{\partial G}\cdot \frac{\partial \phi }{\partial v}={S}_{{\rm{mag}}}\cdot {S}_{{\rm{str}}}\cdot {S}_{{\rm{geo}}}\mathrm{.}$$

## Electrical Properties

**Figure 2:**
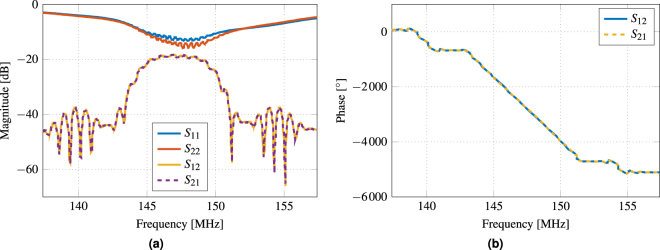
Scattering parameters of the SAW device measured with a vector network analyzer at *B*_bias_ = 0 T. (**a**) The passband of the SAW device around the center frequency of 147.2 MHz offers a 3 dB bandwidth of 4.4 MHz, where the insertion loss is approximately −20 dB. (**b**) The phase decreases virtually linear with a slope of −460 °/MHz.

Figure 2 shows the measured scattering parameters of the fabricated Love wave SAW sensor, which were determined with a vector network analyzer at zero bias flux density *B*_bias_ = 0 T.

Prior to the measurements, the electrical impedance of each port was matched to the system’s impedance of 50 Ω. Hence, the return loss was reduced, slightly different for each port due to component tolerances, between −13 dB and −15 dB in the passband around the center frequency of 147.2 MHz. However, in the same frequency band an insertion loss of about −20 dB was obtained, which is typical for Love wave delay line sensors^28,29^. The total phase angle decreases virtually linear over a frequency range of 7.6 MHz between 143.4 MHz and 151 MHz with a slope of ∂*φ*/∂*f* = −460 °/MHz corresponding to a time delay of *τ*_SAW_ = 1/(2*π*) ⋅ ∂*φ*/∂*f* = 1.28 μs. Due to its insertion loss, the SAW device is only usable as a sensor within the 3 dB bandwidth from 145 MHz to 149.4 MHz since the signal-to-noise ratio (SNR) decreases with increasing attenuation^30^.

## Magnetic Properties

The origin of the magnetic field-induced phase shift can be directly attributed to the change of effective stiffness constants of the ferromagnetic film. The effective stiffness constants are functions of magnetization, and therefore depend on the direction and amplitude of the external magnetic field *H* and the magnetic easy axis (EA) orientation. In the following, a magnetomechanical mean field model is used to quantify the magnetic properties of the ferromagnetic film and derive consequences for sensor optimization later on.

The magnetic mean field model is based on an ensemble of simple uncoupled Stoner-Wohlfarth particles. For each particle, the static equilibrium magnetization *M*_0_ is obtained by minimizing the internal energy density. A distribution of the effective anisotropy energy density *K*_eff_ and of the easy axis orientation *γ*_*i*_ is used to approximate the magnetization distribution. A standard distribution for *K*_eff_ with standard deviation *δ* and a van Mises distribution with dispersion parameter *κ* for the dispersion of the effective easy axis is assumed. Hence, an ideal magnetic film is described by a small *δ* and a large *κ*. Distributions may arise from local stray fields, local stresses, or an intrinsic distribution of the easy axis, e.g., from the deposition process. For the simulation of magnetic hysteresis curves, the magnetization of all particles is averaged. Following the approach by Mater^31^, the magnetoelastic wave results in a small dynamic perturbation, which justifies an expansion of the enthalpy density around the static equilibrium. The effective stiffness tensor *C* can be described by a constant, magnetization-independent part *C*_m_ and a superposed magnetization-dependent part Δ*C*. The stiffness correction tensor Δ*C* is then obtained from the derivatives of the enthalpy density with respect to the magnetization orientation. These derivatives depend on *M*_0_ and are obtained from the magnetic mean field model. For our specific axis configuration, this approach reduces to the Zhou model as described previously^26^. Because *M*_0_ is a function of space, the effective stiffness is also considered as a local property and underlies a spatial distribution. As input for the mechanical model, these values are averaged.

**Figure 3:**
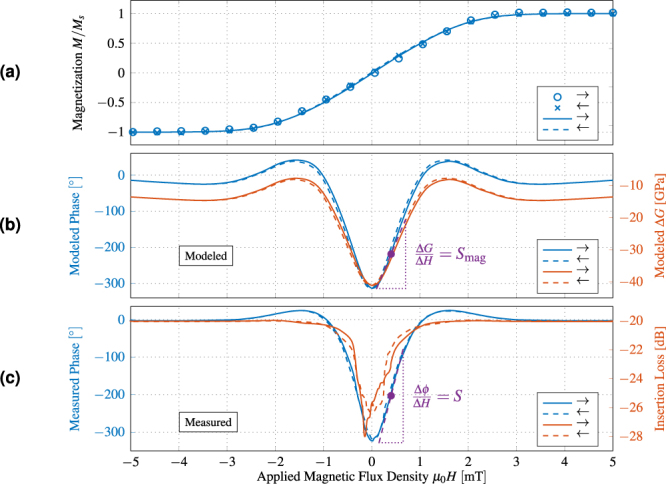
Characterization and modeling of the SAW device. (**a**) Measured magnetization curve of the SAW device using MOKE magnetometry (dot and cross symbols) together with the modeled magnetization curve (solid and dashed lines). (**b**) Modeled Δ*G* with a minimum of −30 GPa at 0 mT. This corresponds to 55% change relative to the value at fixed magnetisation *G*_m_. With these data, the change of phase is calculated using the mechanical model. (**c**) Measured phase shift of the SAW device per magnetic field and corresponding insertion loss at the center frequency of 147.2 MHz. All arrows in the legends represent the direction of the magnetization process starting from a magnetically saturated state.

To obtain the structural sensitivity *S*_str_ from the derived magnetic-field-dependent shear modulus, the concentration effect of the wave at the surface due to the changed shear modulus has to be considered. Hence, an FEM model is used to calculate the wave properties. The propagation region depicted in the schematic of Fig. 1a consists of a horizontally layered stack of a quartz substrate, a SiO_2_ guiding layer, and a magnetostrictive layer. Assuming isotropic material parameters, the structure may be approximated by only considering the *z* dependence. The solution to the one-dimensional problem is given by the following differential equation for the displacement *U*(*k*, *z*, *ω*)^32^

5 $$\frac{d}{dz}[G(z)\,\frac{dU}{dz}]=[{k}^{2}\,G(z)-{\omega }^{2}\,\rho (z)]\,U(k,z,\omega )\,$$

with the shear modulus *G*, the wave vector *k*, and the density *ρ*. The anisotropic parameters of the ST-cut quartz substrate and the magnetostrictive layer are reduced to isotropic values for this simulation. The neglected stress components are lower by at least a factor of 10 than the considered component. Therefore, the error made by this simplification does not influence the general behavior as shown by the small deviation between measurement and simulation in Fig. 3. Eq. (5) is solved numerically with the FEM software package Comsol Multiphysics. To obtain the structural sensitivity *S*_str_ at the operating point, the first derivative of the simulated wave velocity *v* is calculated at the maximum of the sensitivity *S*_mag_ of the material. The model results are shown in Fig. 1b for a variation of the guiding layer thickness.

Hard axis magnetization curves of the complete ferromagnetic film were recorded using large-view magneto-optical Kerr effect (MOKE) microscopy^33^. The measured data are shown in Fig. 3a as dot and cross symbols. The magnetic model was fitted to the MOKE data to extract the magnetic material parameter (solid and dashed lines). For the fit, an effective distribution of the easy axis of magnetization of *κ* = 5500 is used. With *δ* = 300 J/m^3^ a broad effective anisotropy distribution is described around a mean value of *K*_eff_ = 1.3 kJ/m^3^. As a result, a magnetoelastic coupling factor of *b* ^*γ*,2^ = −12.7 MPa is obtained, corresponding to a positive saturation magnetostriction. This value is slightly larger compared to previously reported results for FeCoSiB thin films^13^.

In Fig. 3b the resulting function of Δ*G* is shown. It resembles a V-shaped curve, which is consistent with the results of other authors but exceeds the maximum absolute change of *G* by a factor of about two^26^. Note that Δ*G* < 0 MPa for all values of the magnetic field, as a direct consequence of the averaging procedure. From Δ*G* the maximum slope at a magnetic bias field of μ_0_ *H* = 0.4 mT is directly obtained, resulting in a magnetic sensitivity of *S*_mag_ = 36.8 GPa/mT.

From the variation of shear modulus, the phase is calculated as a function of *H* using the mechanical model. For Love waves, the dominant component of *C* is expected to be the shear modulus *G*. Consequently, the functions of the modeled phase and shear modulus are of similar overall appearance, as shown in the figure. In Fig. 3c the measured phase shift of the SAW is plotted together with the insertion loss. This loss can be attributed to periodic changes of the magnetization by magnetoelastic coupling to the surface wave. The magnetization changes lead to the formation of eddy currents that dissipate the energy by Joule heating. An identical maximum phase shift of 340° is obtained and a similar general behavior for measurement and simulation is confirmed. From the slope of the measured phase we obtain a maximum sensitivity of *S* = 504 °/mT, which is in agreement with *S* as calculated from the modeled data with an accuracy about 10%. Deviations are expected to arise from local stress anisotropies or stray fields that result in slightly different distribution functions.

## Sensor System

**Figure 4:**
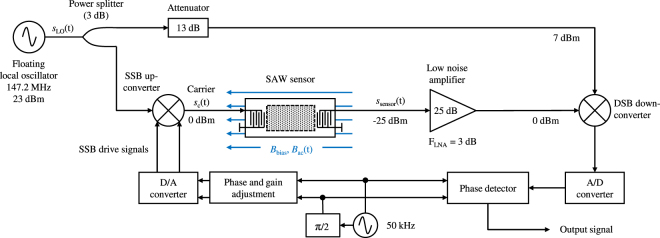
Readout circuit with inherent phase noise suppression. A low phase noise signal of a numerically controlled oscillator (NCO) at 50 kHz is transposed to the operating frequency range of the SAW device and received using a floating local oscillator, which eliminates the phase noise of the local oscillator. To avoid distortions due to an undesired sideband, a signal sideband upconverter is used. Phase detection is achieved in the digital domain after analog-to-digital conversion.

**Figure 5:**
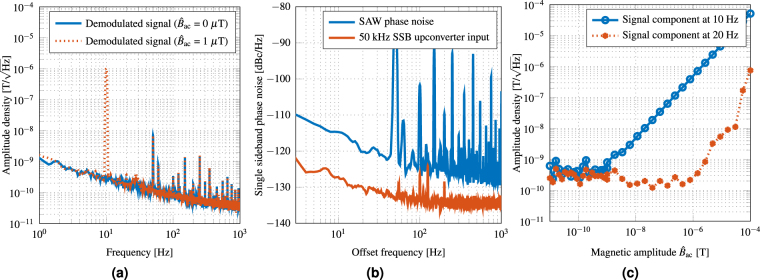
SAW device noise and linearity characteristics. (**a**) Equivalent magnetic noise floor of the demodulated output signal shows the frequency dependent LOD. At 10 Hz an equivalent magnetic noise of 250 pT/$\sqrt{{\rm{Hz}}}$ and at 100 Hz a value of 80 pT/$\sqrt{{\rm{Hz}}}$ are achieved. The LOD is degraded by 1/*f* noise at low frequencies. (**b**) The measured single sideband phase noise of the 50 kHz SSB upconverter input signal reveals a 10 dB lower phase noise value at 10 Hz than the contribution of the SAW sensor itself. (**c**) The response of the phase demodulated sensor signal increases linearly over a large range of magnetic amplitudes ${\hat{B}}_{{\rm{ac}}}$. The linearity (at *f*_ac_) as well as the nonlinearity represented by the first harmonic (at 2*f*_ac_) is plotted. From amplitudes of 100 μT to approximately 250 pT, a linear response is measured. The interception point of the linearity line and the progression of the first harmonic is not reached for magnetic amplitudes ${\hat{B}}_{{\rm{ac}}} < 100\,{\rm{\mu }}{\rm{T}}$.

The electronic readout circuit is depicted in Fig. 4. A sinusoidal carrier signal *s*_c_(*t*) is injected into the sensor with a frequency *f*_c_ within the SAW device’s passband. Through the magnetic field-dependent travelling time and, thus, the phase shift of the carrier, the magnetic flux density *B*_ac_(*t*) can be measured. The sensor’s output signal is given by

6 $${s}_{{\rm{s}}{\rm{e}}{\rm{n}}{\rm{s}}{\rm{o}}{\rm{r}}}(t)\propto \cos (2\pi {f}_{{\rm{c}}}t+S\cdot {B}_{{\rm{a}}{\rm{c}}}(t)\cdot {\mu }_{0}^{-1}+{\varphi }_{{\rm{c}}}(t)+{\varphi }_{{\rm{S}}{\rm{A}}{\rm{W}}}(t)),$$

where *S* is the sensitivity defined in Eq. (4). The measurement results in *S* = 264°/mT which is about a factor of two smaller than what was derived from Fig. 3c. However, the deviation is within the variation between the various tested samples. The measurement is impaired by the phase noise *ϕ*_c_(*t*) of the carrier signal and by random phase fluctuations of the SAW device *ϕ*_SAW_(*t*). To minimize the phase noise contribution of the sensor electronics, the carrier *s*_c_(*t*) is derived from the output of a numerically controlled oscillator (NCO) at 50 kHz (phase noise at −128 dBc/Hz at an offset frequency of 10 Hz, cf. Figure 5b), which is upconverted to the SAW device’s passband by means of a single sideband (SSB) upconverter and a local oscillator *s*_LO_(*t*). The single sideband upconverter suppresses an undesired sideband which would fall into the passband of the SAW device. Otherwise the phase measurement after the downconversion process would be distorted. Sufficient sideband suppression is achieved by adjustment of amplitude and phase of the SSB drive signals in the digital domain. The sensor output signal *s*_sensor_(*t*) is amplified and downconverted to the original frequency of 50 kHz by means of a double sideband mixer (DSB) using the same local oscillator *s*_LO_(*t*). Thus, the phase noise *ϕ*_c_(*t*) of the carrier is largely suppressed. The degree of suppression depends on the delay time of the SAW device *τ*_SAW_^34^. For the Love wave SAW sensor with typical delay times between 1 μs and 2 μs presented here, LO phase noise is reduced by approximately 80 dB for an offset frequency of 10 Hz and by approximately 60 dB for an offset frequency of 100 Hz, respectively^35^. The local oscillator utilized here has a phase noise of −80 dBc/Hz and −95 dBc/Hz, respectively, and is therefore — after downconversion — well below the phase noise of the low-frequency 50 kHz signal. Final phase detection is carried out digitally. The bidirectional conversion between the analog and the digital domain is performed by high resolution 24-bit converters at a sample rate of 192 kHz.

Due to the chosen operating frequency of 50 kHz, the bandwidth of the sensor system is limited to this frequency. In general, the SAW bandwidth permits a system bandwidth up to 2 MHz by using a higher operating frequency. However, other limiting factors can appear. A trade-off between the system noise due to the NCO and the bandwidth must be found for the chosen application.

In Fig. 5a the equivalent magnetic noise floor achieved with the sensor system is shown. It was measured inside a magnetically and mechanically shielded chamber^36^. At 10 Hz and 100 Hz equivalent magnetic noise levels of 250 pT/$\sqrt{{\rm{Hz}}}$ and 80 pT/$\sqrt{{\rm{Hz}}}$, are achieved, respectively. These values are well within the 100 pT/$\sqrt{{\rm{Hz}}}$ range of thin-film ΔE-effect sensors^20^, but the bandwidth is far higher.

Obviously, the measured equivalent magnetic noise density in Fig. 5a is degraded by 1/*f* noise in the low-frequency regime. This contribution originates from the SAW sensor itself, which is a common phenomenon^37–39^. However, the origin of this noise contribution is presently not well understood^39^. With the same method as proposed by Baer^39^ the phase noise of the SAW sensor presented here was measured for *B*_bias_ = 0 T after the measurement setup was carefully calibrated^40^. The result is shown in Fig. 5b. Power line spurs and subharmonics can be seen and must be disregarded when analyzing the data. The measurement reveals a single sideband phase noise level of −115 dBc/$\sqrt{{\rm{Hz}}}$ for an offset frequency of 10 Hz. The value is 10 dB^40^ to 20 dB^38^ higher than phase noise densities previously reported in the literature. However, these values are not directly comparable due to different structures, delay times, and operating frequencies of the various SAW devices. Further investigation with respect to the phase noise properties of Love wave SAW sensors is required. A lower phase noise would directly result in an improved LOD.

In many applications the dynamic field range is of extreme importance. Biomagnetic measurements without magnetic shielding, which is, e.g., mandatory for any long-term monitoring, would require differential measurements to extract the biomagnetic signals from the million-times higher noise background. Thus, a dynamic range of at least 120 dB is required for these measurements. In the case of current sensors, such as for electro-mobility applications, there is again the need to measure both very small leakage currents and very high currents under full load using the same sensor. Figure 5c shows the linear response of the phase demodulated signal of the Love wave SAW sensor. Across a range of 120 dB (approximately from 100 pT to 100 μT) linear behaviour is achieved. The intercept point of the linear response at *f*_ac_ = 10 Hz and the nonlinear response, measured by the first harmonic response at 2*f*_ac_ = 20 Hz, is not reached for magnetic amplitudes ${\hat{B}}_{{\rm{ac}}} < 100\,{\rm{\mu }}{\rm{T}}$. Hence, the sensor is suitable for unshielded measurements since it would not be saturated by earth’s magnetic field.

## Sensitivity Improvement

**Figure 6:**
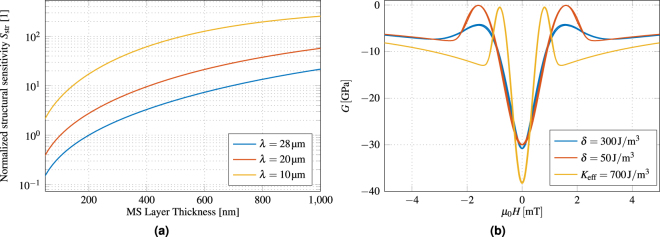
Modeling of potential sensitivity improvements. (**a**) Normalized structural sensitivity *S*_str_ for different magnetostrictive (MS) layer thicknesses and different periodicities of the IDTs. For thicker MS layers as well as for lower wavelengths the sensitivity is improved. (**b**) Calculated change of shear modulus ΔG for our sensor (*δ* = 300 J/m^3^), a strongly reduced anisotropy dispersion (*δ* = 50 J/m^3^), and a reduced anisotropy dispersion with additionally reduced mean anisotropy energy density (*K*_eff_ = 700 J/m^3^).

To improve the sensitivity of the Love wave magnetic field sensor described here, various strategies can be followed. Because the readout electronics are not the dominating noise of the sensor system, the sensitivity of the sensor has to be increased or its intrinsic noise level has to be lowered to achieve a higher SNR. A first approach in order to increase the sensitivity is to improve the structural sensitivity *S*_str_, which could be achieved either with a thicker magnetic layer, a thicker guiding layer, or a shorter wavelength. According to Fig. 6a the sensitivity can be increased significantly for a magnetostrictive layer thickness of 400 nm by a factor of 3 to 20 by reducing the wavelength to 20 μm or 10 μm, respectively. A second measure to improve the structural sensitivity would be thicker magnetostricive layers, although this might have conflicting influence on the magnetic properties of the film. A reduction of the wavelength by a factor of 2.8 would also multiply the geometric sensitivity by the same factor. However, all these measures might potentially increase the insertion loss of the sensor. A compromise between delay time and insertion loss has to be made. The insertion loss could potentially be reduced by structuring the magnetic film with isolating layers to reduce eddy currents^27^.

A third approach is to optimize the magnetic properties of the magnetostrictive layer to increase the magnetic sensitivity *S*_mag_, especially the key parameters of the mean anisotropy energy density *K*_eff_ and the effective anisotropy distribution *δ*. In Fig. 6b the calculated change Δ*G* of shear modulus is plotted for the current sensor parameters (*δ* = 300 J/m^3^), a strongly reduced anisotropy distribution (*δ* = 50 J/m^3^) and an additionally reduced mean anisotropy energy density (*K*_eff_ = 700 J/m^3^). From the first two datasets of Fig. 6b, the influence of *δ* is evident. A reduced dispersion of *K*_eff_ results in an increase of the two maxima to Δ*G* ≈ 0 GPa. Additionally, decreasing *δ* reduces the curvature at about 2.5 mT, which becomes a discontinuity for zero *δ*. Interestingly, the region around 0 mT between the two maxima is barely influenced by the distribution. The magnetic working point for the sensor is set, where the model predicts identical maximum magnetic sensitivities for both distributions. Because smaller *K*_eff_ are prone to static stresses and local stray fields, they are expected to be accompanied by larger *δ*. Hence, the current configuration is advantageous if small mean anisotropy energy densities can be achieved. As an example, the third dataset (*K*_eff_ = 700 J/m^3^) shows the change of shear modulus with respect to magnetic field for *K*_eff_ additionally reduced by a factor of about two, which yields a corresponding increase in sensitivity. If *K*_eff_ cannot be reduced, the easy axis can be tilted by 90 degrees relative to the propagation direction of the wave. In this configuration we expect to approach a singularity instead of a discontinuity for vanishing *δ* ^17^. As a result, the sensitivity is dominated by *δ* instead of *K*_eff_. Initial estimations yield to an improvement of the magnetic sensitivity *S*_mag_ by a factor between 2 and 7, depending on *δ*. Consequently, future sensor designs must make a compromise between small *K*_eff_ and small *δ* depending on the axis configuration of the magnetic film. Such arguments are similarly valid for an angular distribution of the easy axis. To allow bias field-free operation, the magnetic layer could be biased using exchange coupled multilayers, as has been demonstrated for other magnetostrictive sensors^41^.

A fourth method to increase the SNR could be the allocation of several carrier signals within the transmission bandwidth of the sensor, provided that the respective noise signal are uncorrelated. The sensor offers enough bandwidth for approximately 50 carriers, spaced 100 kHz apart. The signals of these carriers could be processed separately and, thus, the noise should be reduced by $\sqrt{50}\approx 7$ according to Reermann *et al*.^42^. Thermal cross sensitivity could be reduced with an uncoated delay line as a reference sensor to improve DC stability. A significant improvement by two orders of magnitude of the overall sensitivity might be achieved by combination of the proposed measures. Nevertheless, a higher sensitivity only increases the signal strength and the goal is always to improve the signal to noise ratio. It is possible, that trade offs will have to be made to respond to potential increase of noise.

## Conclusion

Love wave SAW delay lines present an effective means to utilize the magnetoelastic effect in thin films for magnetic field sensing. A comprehensive study of the complete sensor system is presented, including measurements and simulations of the electrical, mechanical, and magnetic properties. With a detection limit of ≈100 pT/$\sqrt{{\rm{Hz}}}$ over a large bandwidth of 50 kHz and a dynamic range larger than 120 dB, the sensor system is a promising addition to existing sensor concepts. Additionally, there is the potential to greatly increase the bandwidth up to the MHz-range. Significant improvements of sensitivity can also be expected by further perusing the Love wave concept of wave confinement with thicker and magnetically softer layers, or higher SAW frequencies.

## Methods

### Sensor fabrication

The Love wave sensors are based on 500 μm ST-cut quartz wafers (42° 45′ Y-cut) for SAW applications. All layers, except the SiO_2_ guiding layer, are deposited by magnetron sputtering using a von Ardenne CS730 S sputtering system. Cr and Au are deposited by DC sputtering. The magnetostrictive layer is deposited by RF sputtering with a target composition of (Fe_90_Co_10_)_78_Si_12_B_10_ while applying a magnetic field of 100 Oe. Lift-off of the magnetostrictive layer is performed with the negative photoresist AZ nLof 2070 from MicroChemicals. For ion-beam etching of the IDT structures, the positive photoresist AZ 1518 also from MicroChemicals is used. The SiO_2_ layer is deposited by a PECVD process with a SENTECH SI 500 PPD tool and afterwards structured by an ICP-RIE etching process.

### Magnetic measurements

The magnetic measurements of Fig. 3 are performed at a temperature of 21 °C in the center of a pair of 10 cm Helmholtz coils. The magnetically induced phase shift is averaged from 25 measurements between 148 and 149 MHz with a lock-in amplifier (Zurich Instruments UHFLI) to compensate for small non-linearities in the device relation of phase and frequency. The non-linearities are attributed to electrical feedthrough and also cause the fine ripple in the scattering parameters.

### Model and Material Properties

With regards to the magnetic model, the internal energy density *h* is minimized for each Stoner-Wohlfarth particle. It can be described using Einstein notation by^15,43^

7 $$h={K}_{{\rm{eff}}}{\sin }^{2}[\arccos ({\alpha i^{\prime} }_{}{\gamma i^{\prime} }_{})]-{\mu }_{0}H{M}_{{\rm{s}}}{\alpha i^{\prime} }_{}{\beta i^{\prime} }_{}\mathrm{.}$$

Here *α*_*i*_′,*β*_*i*_′ and *γ*_*i*_′ denote the direction cosines of the equilibrium magnetization, the applied magnetic field with amplitude *H*, and the easy axis orientation vector with respect to the coordinate axes. *K*_eff_ is the effective anisotropy energy density constant of first order. All clamping effects and initial stress contributions are taken into account by *K*_eff_. For the saturation magnetization *M*_*s*_, we used μ_0_ *M*_*s*_ = 1.5 T^44^. Furthermore, we assume in-plane magnetization. The magnetoelastic coupling coefficient *b* ^*γ*,2^ is defined according to Callen and Callen^45^.

The mechanical model for the Love waves is described by Eq. (5) and is calculated using Comsol Multiphysics with the following boundary conditions: the bottom interface is clamped (displacement *u* = 0) and the top interface is a free surface (stress *σ*_12_ = 0). At the layer interfaces, continuity of displacement and stress is required. The anisotropic parameters of the ST-cut quartz substrate are reduced to the isotropic values *G*_Quartz_ = 49.2 GPa, *ρ*_Quartz_ = 2650 kg/m^3^ for this simulation. For the mechanically isotropic magnetostrictive layer, we use *E*_MS_ = 150 GPa, *ν*_MS_ = 0.38 and *ρ*_MS_ = 7250 kg/m^3^, which results in *G*_MS_ = 54.3 GPa for the shear modulus at fixed magnetization. For the amorphous, isotropic guiding layer, the following parameters are used: ${E}_{{{\rm{SiO}}}_{{\rm{2}}}}=77.6\,{\rm{GPa}}$, ${\nu }_{{{\rm{SiO}}}_{{\rm{2}}}}=0.1638$, and ${\rho }_{{{\rm{SiO}}}_{{\rm{2}}}}=2200\,{\mathrm{kg}/{\rm{m}}}^{3}$. For the magnetic sensitivity, a value of *S*_mag_ = 36.8 GPa/mT was determined from the modeled change of *G* depicted in 3b at the operating point of μ_0_ *H* = 0.4 mT. The structural sensitivity is depicted in 1b with a value of *S*_str_ = 1.07 $\frac{{\rm{m}}/{\rm{s}}}{{\rm{G}}{\rm{P}}{\rm{a}}}$. For the calculation of *S*_geo_ the following parameters were used: a delay line length *l* = 3.8 mm, a frequency *f* = 147.2 MHz and a wave velocity of *v* = 4220 m/s. The overall sensitivity derived from model and simulations is *S* = 450°/mT.

### Electrical properties

To measure the scattering parameters of the Love wave SAW sensor, the vector network analyzer E8361A from Agilent Technologies is used. Prior to the measurements, both ports of the sensor are matched to 50 Ω by means of a parallel capacitor of 22 pF and a series inductor of 202 nH (as seen from the 50 Ω ports into the sensor ports).

### Readout circuit

The readout circuit is mainly composed of various components from Mini-Circuits. The SSB upconverter consists of a 2-way-90° power splitter ZMSCQ-2-180+ at the input and a 2-way-0° combiner ZMSC-2-1W+ at the output. In-between, two level 17 mixers ZAD-1H+ perform the frequency conversion. The downconversion is realized by a level 7 mixer ZP-3+ after the output signal of the SAW is amplified by a low noise amplifier ZFL-1000LN+. For the floating LO an SMBV100A vector signal generator from Rohde & Schwarz is utilized. The bidirectional conversion between the analog and the digital domain is performed by a high resolution 24-bit converter RME Fireface UFX at a sample rate of 192 kHz. The digital signals are processed in the in-house built real-time framework KiRAT (Kiel Real-time Application Toolkit) as well as in MATLAB. The sinusoidal oscillator signals at 50 kHz, ahead referred to as NCO, are calculated in real-time by means of the CORDIC trigonometric computing technique^46^.

### Equivalent magnetic noise floor

The equivalent magnetic noise floor is measured with the use of a calibrated solenoid. With the calibration information and the measured transfer function a scaling-based equalization on a scaling is performed in the time domain after the demodulation process with the help of an in-phase and quadrature (IQ) approach. Due to this processing, a signal with the unit Tesla equivalent to the applied magnetic field is generated. The length of the recorded signal is set to 30 s.

To determine the power spectral density (in units of T^2^/Hz), Welch’s method^47^ is used. Based on the utilized flattop window and the FFT order of 8 times the sample rate, the effective noise bandwidth (ENBW) is set to a value of 0.47 Hz, which is taken into account in the power spectral density estimation. The overlap is set to three quarters of the FFT order. To obtain the amplitude spectral density (in units of ${\rm{T}}/\sqrt{{\rm{Hz}}}$), which represents the magnetic noise floor, the square root is taken.
